# Comparison of the South African Spondaic and CID W-1 wordlists for measuring speech recognition threshold

**DOI:** 10.4102/sajcd.v62i1.97

**Published:** 2015-06-26

**Authors:** Tanya Hanekom, Maggi Soer, Lidia Pottas

**Affiliations:** 1 Department of Speech-Pathology and Audiology, University of Pretoria, South Africa

## Abstract

**Background:**

The home language of most audiologists in South Africa is either English or Afrikaans, whereas most South Africans speak an African language as their home language. The use of an English wordlist, the South African Spondaic (SAS) wordlist, which is familiar to the English Second Language (ESL) population, was developed by the author for testing the speech recognition threshold (SRT) of ESL speakers.

**Objectives:**

The aim of this study was to compare the pure-tone average (PTA)/SRT correlation results of ESL participants when using the SAS wordlist (list A) and the CID W-1 spondaic wordlist (list B – less familiar; list C – more familiar CID W-1 words).

**Method:**

A mixed-group correlational, quantitative design was adopted. PTA and SRT measurements were compared for lists A, B and C for 101 (197 ears) ESL participants with normal hearing or a minimal hearing loss (<26 dBHL; mean age 33.3).

**Results:**

The Pearson correlation analysis revealed a strong PTA/SRT correlation when using list A (right 0.65; left 0.58) and list C (right 0.63; left 0.56). The use of list B revealed weak correlations (right 0.30; left 0.32). Paired sample *t*-tests indicated a statistically significantly stronger PTA/SRT correlation when list A was used, rather than list B or list C, at a 95% level of confidence.

**Conclusions:**

The use of the SAS wordlist yielded a stronger PTA/SRT correlation than the use of the CID W-1 wordlist, when performing SRT testing on South African ESL speakers with normal hearing, or minimal hearing loss (<26 dBHL).

## Introduction

### Background

South Africa is a country with a distinctly unique amalgamation of languages, dialects, cultures and linguistic communities (Swanepoel, 2006). Audiologists in South Africa face a predicament when conducting speech audiometry tests on English Second Language (ESL) speakers. The majority of audiologists registered in South Africa are English- or Afrikaans-speaking individuals (Penn, Frankel, Watermeyer & Muller, 2009) who are unlikely to speak an African language (Khoza, Ramma, Mophosho & Moroka, 2008). This is in stark contrast to the demographics of South Africa, where the home language of 77% of the population is an African language (Statistics South Africa, 2012). Only 9.6% of South Africans speak English as their first language (Statistics South Africa, 2012).

However, a cycle perpetuates wherein English remains the dominant language in South African society and the majority of the population (between 32% and 69%; there are broad ranges in the statistical estimates) uses English as one of their multiple languages, particularly in urban areas (Minow, 2010). English is used extensively in South Africa in education, law, government, news broadcasts, business, commerce, the army and parliamentary debate (Alexander, 2000; Minow, 2010). English is the language of learning and teaching (LoLT) for more than 90% of South African learners (De Wet, 2002). It is within this context that audiologists in South Africa are aiming to provide quality audiological assessments to the population.

Audiology uses various measures to determine hearing function, including pure-tone testing and speech audiometry. The measurement of the speech recognition threshold (SRT) is a speech audiometry test that relies on the participant's recognition of familiar spondaic words from a closed set. SRT is defined as the lowest intensity at which the spondaic (spondee) words are identified correctly 50% of the time (Martin & Clark, 2003). Spondaic words are made up of bi-syllabic words, typically nouns, with equal stress placed on each syllable, such as ‘sunset’ (Gelfand, 2009). In 1947, Hudgins, Hawkins, Karlin and Stevens (1947) first developed a list of spondaic words at the Harvard Psycho-Acoustic Laboratories (PAL). Following the development of the PAL wordlists, the words were evaluated for homogeneity of audibility, within ±2 dB for mean recognition thresholds (Gelfand, 2009). Six words were excluded from the original 42 spondaic words because of a lack of homogeneity (McArdle & Hnath-Chisolm, 2009); and the remaining 36 were recorded, thus forming the published Central Institute for the Deaf Auditory Word List W-1 (CID W-1) (Hirsh et al., 1952). Many of the words, such as ‘inkwell’, ‘drawbridge’ and ‘horseshoe’, are specific to American-English and are not necessarily familiar to South African ESL speakers (Ramkissoon, Proctor, Lansing & Bilger, 2002).

### Literature review

Familiarity of spondaic words is ‘arguably one of the most important aspects to consider when choosing stimuli because it helps ensure the validity of the test’ (Nissen, Harris, Jennings, Eggett & Buck, 2005, p. 391). Familiarity means that the participant is exposed frequently to the word, both using and hearing the word in a social setting, as well as being aware that the word is part of the test items (Ramkissoon, 2001). Word familiarity is dependent on the individual, but certain words are more familiar than others in a socio-linguistic group (Brandy, 2002). Familiarity ensures that the auditory threshold of the participant is measured, not their vocabulary (Ramkissoon, 2001). Familiarity improves one's recognition of words and allows for improved test performance, particularly when listening to moderately degraded acoustic signals, as in SRT testing (Sreedhar, Venkatesh, Nagaraja & Srinivasan, 2011).

Several wordlists have been developed in African languages in South Africa, but they are generally not formally standardised and are not yet readily available in recorded format (Khoza et al., 2008; Khoza-Shangase & Mokoena, 2014). One of the recognised shortfalls of these studies was that the participants were all tertiary students enrolled at a university where the language of instruction was in English (Khoza et al., 2008). In addition, the words were presented by a Tswana-speaking individual, which represents a small portion of audiologists in South Africa (Khoza et al., 2008; Penn et al., 2009). There are very few audiologists who are proficient in African languages, which may result in significant errors of production (Khoza et al., 2008). Clinicians should be proficient in the language of the test in order to ensure accuracy in administration and scoring (Ramkissoon, 2001). The mismatch between the number of audiologists who speak an African language and the population of South Africa means that the use of African wordlists is not a practical solution at this time in South Africa.

On the contrary, all audiologists are likely to use English as one of their multiple languages (Swanepoel, 2006) and the majority (up to 69%) of the population speaks English as one of their multiple languages, particularly in urban areas (De Klerk, 1999). The use of an English wordlist that is familiar to all South Africans who use English as one of their multiple languages may be a good solution. One of these alternative wordlists may be the South African Spondaic (SAS) wordlist which was developed by the author (Durrant, 2006).

In 2006, the author generated a list of English spondaic words following Hudgins et al.'s (1947) guidelines. These words were selected from common everyday South African English words (Durrant, 2006). The words were determined to be structurally balanced in terms of the consonant-vowel-consonant (CVC) structure of each syllable (CVC-CVC) with the assistance of a linguistics lecturer at the university (Durrant, 2006). To determine the familiarity of the words, a sample of 387 participants (both English first-language and ESL speakers) from the Gauteng province, who represented the South African population in terms of first-language, additional spoken languages, age, gender, occupation and education levels, rated the spondaic words in terms of familiarity, through self-report, on a three-point scale (Durrant, 2006). The 18 highest-rated words were determined to be the more familiar spondaic words amongst the South African population and may be known as the South African Spondaic (SAS) wordlist. For the purpose of the current research, this wordlist may be viewed as list A in the Appendix, Table 1-A1 (Durrant, 2006). Permission was granted by the relevant university to use this wordlist for the current study.

The familiarity of the existing CID W-1 wordlist (Hirsh et al., 1952) was rated at the same time. The CID W-1 words were grouped into list B (least familiar) and list C (more familiar), according to the same rating scale. Clinically, this may have implications if the ‘less familiar’ words were excluded when testing ESL speakers. However, the exclusion of certain words would reduce the set size of a list and is, therefore, not recommended (Ramkissoon & Khan, 2003).

The development of other wordlists historically involved the informal generation of wordlists which were non-standardised prior to testing (Khoza et al., 2008; Nissen et al., 2005; Sreedhar et al., 2011). The words were rated for familiarity (Nissen et al., 2005; Sreedhar et al., 2011) and inappropriate words were excluded from the wordlists. This procedure was similar to the procedure followed previously by Durrant (2006).

### Research objectives

The current study aims to determine the correlation of the SRT obtained with the SAS wordlist, when compared to pure-tone average (PTA). The validation of pure-tone thresholds is conducted by comparing SRT to the average pure-tone thresholds at 500 Hz, 1000 Hz and 2000 Hz (Brandy, 2002). The PTA/SRT agreement should be in close agreement. Clinically, literature specifies that PTA and SRT should be within 6 dB of one another to indicate good correlation and reliability from test results (Brandy, 2002). This may be referred to as the PTA/SRT relationship. Should there be a larger difference between PTA and SRT, the validity of the measurements comes into question, possibly indicating non-organic hearing loss (Khoza-Shangase & Mokoena, 2014).

The research question was: ‘Which list (CID W-1 or SAS wordlist) yields the strongest PTA/SRT correlation when testing a group of South African ESL participants?’

## Research method and design

### Research aim

The primary aim of this study was to compare the SAS wordlist and the CID W-1 wordlist for measuring SRT when testing a group of South African ESL participants.

### Specific objectives

To determine and compare the PTA/SRT correlation of ESL participants when using the SAS wordlist (list A) and the CID W-1 wordlist (lists B and C).

### Design

A mixed-group correlational design within a quantitative paradigm was adopted. The design contained both between-participants and within-participants designs. A mixed-group design studies one independent variable (SAS wordlist, list A) with a within-participants design, as well as other independent variables (two conditions, lists B and C) with a between-participants design (Schiavetti & Metz, 2006).

### Participants

#### Sample and sampling

Participant selection was based on purposive sampling, where 101 (197 ears) normally-hearing ESL employees from the participating hospital volunteered to participate in the study. The management of the participating hospital granted permission to conduct the study, once they were informed about the implications on the employees’ wellbeing and time demands. Advertisements for participants included the use of posters, word of mouth and announcements distributed within the hospital. The participation in the research required each participant to be present for a 15-minute interval.

Participants needed to meet the following criteria in order to take part in the study:

- **Age:** Participants had to be older than 18 years of age in order for informed consent to be obtained without parental consent (Coyne, 2010). There was not an upper age limit, as factors such as auditory processing related to age were not considered to be significant.
- **Language:** Participants had to be ESL speakers, with a speaking knowledge of English. ESL speakers may be considered to use English as a second language, with various degrees of proficiency, in order to communicate with other speakers in a given society. As long as the participant could participate in a basic English conversation regarding their age, education and type of work, by responding appropriately, they were not excluded from the study. As the SAS wordlist is targeted primarily for use with speakers who use English as one of their multiple languages, but not as their first language, the participants had to be ESL speakers.
- **Hearing thresholds:** All participants had to have hearing thresholds within normal limits, or a minimal hearing loss (thresholds <26 dBHL from 250 Hz to 8000 Hz), according to Silman and Silverman's (1991) classification system (Schlauch & Nelson, 2009). Pure-tone testing was conducted by the researcher prior to measuring SRT. Exclusion was ear-specific if hearing loss was present in one ear only, as the data analysis was ear-specific. The criterion of hearing thresholds within normal limits was necessary at this stage in order to eliminate extraneous variables such as degree, type and configuration of hearing loss (Schiavetti & Metz, 2006), as well as the impact of hearing loss on speech audiometry findings. The participants with hearing loss were referred for further diagnostic testing.
- **Employment:** ESL speakers who were employed at the participating hospital were invited to participate in the study. This was primarily for the purpose of convenience sampling. The sample included participants employed as nurses, administrators, managers, cleaners, plumbers, waitresses and security personnel at the hospital. The participants are therefore representative of various groups of individuals, although this might have limitations as the generalisation may be compromised because of the relatively small sample size (Schiavetti & Metz, 2006).

### Procedure

#### The recording of wordlists

The development of the speech audiometry material was described in the introduction. The following section describes the recording of the wordlists for the purposes of this study. The wordlists were recorded in order for test–retest reliability to be preserved in the research process (Schiavetti & Metz, 2006).

The wordlists (lists A, B and C, as seen in the Appendix) were presented with equal stress on each syllable, by a South African, English-first-language female audiologist (the researcher), through the microphone of an Interacoustics diagnostic audiometer (AD229b, last calibrated 6 April 2011). The audiometer is connected to speakers in free-field in a single sound-treated audiology booth (2 m^2^) which was used to eliminate background noise. The words were recorded in the booth with a digital voice recorder (Olympus WS100, serial number 200107495). The digital voice recorder was placed one metre away from the sound source speaker and the spoken words were recorded as various tracks on the digital voice recorder. The spondaic words were recorded without a carrier phrase. The use of a carrier phrase is indicated for supra-threshold testing, but the literature indicates mixed findings with regard to the benefit of using a carrier phrase for threshold testing, as it may be considered time consuming and distracting (Gelfand, 2009).

The recorded words were then analysed in terms of frequency spread, duration and intensity of each syllable within each word, with the software program, Praat (Boersma & Weenink, 2009). The words were normalised to peak at zero decibels (0 dB) and were adjusted to allow for similar frequency spread, duration and intensity of each syllable within and between each spondaic word, so as to allow for maximum homogeneity in terms of audibility (Gelfand, 2009). The words were burned onto a recordable compact disc, to allow for playback via a compact disc (CD) player attached to an audiometer.

Each of the three wordlists were recorded twice, in different randomised orders, to allow for familiarisation with one list and threshold determination with the second list, in order to exclude variables such as order effect (Schiavetti & Metz, 2006).

The audiological testing of participants then followed, using standard protocols for data collection.

### Data collection

The researcher conducted data collection, including case history interviews, otoscopic examinations, immittance testing, pure-tone air-conduction testing and speech audiometry testing. All the procedures were conducted on those participants who had given their informed consent (*n* = 104).

#### Case history interview

Structured interviews were conducted in order to obtain demographic information about the participants’ age, gender, language use, language exposure, subjective verbal self-rating of language proficiency rated as ‘excellent’, ‘good’, ‘fair’ or ‘poor’, audiological concerns, education and occupation.

#### Otoscopic examination

Otoscopic examinations were conducted on each participant, using a handheld Heine mini 3000 otoscope to visually inspect the outer ear. This was done to ensure there was no excess cerumen in the external ear canal and that the tympanic membranes were visible, and appeared healthy (Martin & Clark, 2003).

#### Immittance audiometry

Tympanometry procedures were conducted on each participant, using a Maico MI34 immittance machine, to assess the middle-ear pressure and compliance (Martin & Clark, 2003). A low-frequency probe tone of 226 Hz was used (Martin & Clark, 2003). Recordings were analysed according to Jerger's classification system (1970, as cited in Martin & Clark, 2003): Type A tympanograms were regarded as normal and the other classifications (Types As, Ad, B, C and D) were considered abnormal. Participants were not excluded from the study based on tympanogram results, unless the middle-ear status was significant to contribute to a hearing loss (PTA > 26 dBHL), as this was otherwise considered negligible for the purposes of the research. The participants with outer- or middle-ear pathology were referred for further medical management.

#### Pure-tone air-conduction audiometry

Pure-tone air conduction audiometry was conducted using an Interacoustics diagnostic audiometer (AD229b), using TDH-39 headphones, in a single sound-treated audiology booth in a quiet office. Pure-tone thresholds were measured by presenting tones at each octave point frequency (250, 500, 1000, 2000, 4000, 8000 Hz), using the modified Hughson-Westlake procedure (Martin & Clark, 2003). Those participants who had audiometric thresholds lower than 26 dB from 250 Hz to 8000 Hz were considered to have hearing within normal limits, or a minimal hearing loss. The classification of degree of hearing loss was obtained from Silman and Silverman's (1991) classification system. Pure-tone average (PTA) per ear was calculated from the sum of hearing thresholds at 500 Hz, 1000 Hz, and 2000 Hz, divided by three and rounded off to within one decimal place for each ear (Martin & Clark, 2003). Participants with hearing thresholds outside normal limits were excluded from the study at this point.

Exclusion was ear-specific because of the presence of hearing loss. Only participants who met the specified selection criteria were selected for this portion of the study (*n* = 101).

#### Speech recognition threshold

Speech audiometry was conducted using an Interacoustics diagnostic audiometer (AD229b), in a single sound-treated audiology booth in a quiet office, with a CD player connected via an audio cable to allow for presentation of the recorded wordlists. The recorded words were presented through a CD unit attached to the diagnostic audiometer, using TDH-39 headphones, with both syllables of the spondaic words peaking at zero Volume Unit (VU), on the VU meter, to allow for equal loudness presentation between the words (Gelfand, 2009). The participants were instructed to repeat the words back to the researcher, even if they were unclear or soft.

Each of the wordlists was initially presented at 75 dBHL in one ear, in order to familiarise the participant with the spondaic words prior to testing. This is a supra-threshold level that is not considered uncomfortably loud (Brandy, 2002). Familiarisation with the spondaic words is recommended prior to actual threshold testing. The importance of familiarisation is a well-established concept (Gelfand, 2009). If a participant is not familiarised with the words prior to testing; it may result in SRTs that are 4 to 5 dB poorer than their actual thresholds (Gelfand, 2009). All participants were familiarised with list A (the SAS wordlist). Thereafter, the participants were randomly delegated to either group one or group two. The test participants in group one were similarly familiarised with List B (the less familiar CID W-1 words) and the participants in group two were familiarised with list C (the more familiar CID W-1 words).

Following familiarisation, SRT was determined in each ear using the second recordings of list A, B or C, which had the same content as the first lists, but in a different presentation order, to exclude variables such as order effect. SRT was determined by starting with the presentation of a single word at 40 dBHL, and decreasing in 10 dB steps, presenting one word at each intensity level. When one word was repeated incorrectly, the tester stopped descending and presented three more words at that level. The intensity level was then increased or decreased in 5 dB steps to determine the softest intensity at which 50% of the words were repeated correctly, as first described by Carhart (1946, as cited in Brandy, 2002). The order of presentation of the wordlists was randomised in order to minimise the effects of sequencing (Schiavetti & Metz, 2006). The use of 5 dB steps was favoured over the use of 1 dB or 2 dB steps in the interest of clinical timeliness and brevity. However, for research purposes, 1 dB or 2 dB steps would have been more precise (Brandy, 2002).

#### Data analysis

All the raw data were captured on a spreadsheet to allow for systematic management, interpretation and statistical analysis of the data. A correlation matrix was calculated to measure the strength and direction of the relationship between two variables, namely PTA and SRT for lists A, B and C. List A was also split for groups one and two. A Pearson correlation analysis was conducted (Howell, 2009). To determine if the correlation was statistically significant, a statistical test was conducted to determine the probability value (*p*-value) (Howell, 2009).

A correlation relationship may be considered positive if the correlation occurs in the same direction. The strength of the correlation relationship is determined according to the correlation coefficient: a correlational strength of +0.50 to +1.00 is considered a positive, moderate-to-strong correlation; a correlational strength of +0.50 is considered a positive, moderate correlation; and a correlational strength from 0.00 to +0.50 is considered a positive, weak-to-moderate correlation (Schiavetti & Metz, 2006).

#### Ethical considerations

Ethical clearance (reference number 29360979) was obtained from the University of Pretoria Postgraduate Research and Ethics Committee of the Faculty of Humanities prior to the commencement of the study. Approval to conduct the study was obtained from all relevant authorities and departments prior to the conduction of the study. Informed consent was obtained from every participant prior to being included in the study; and participants were issued with a written information sheet about the nature, purpose and risks of the study, which was also explained verbally. Confidentiality and anonymity were ensured by replacing the names with research code numbers (Schiavetti & Metz, 2006).

## Trustworthiness

### Reliability and validity

Testing took place in a sound-proof booth to ensure there was no interference from background noise (Schiavetti & Metz, 2006). Instructions to participants were clear and consistent. The equipment used had undergone its annual calibration at the time of data collection. Biologic calibration was regularly performed and the VU meter was set according to the recorded material.

## Results

Following pure-tone testing, 101 participants (197 ears: 100 right ears, 97 left ears) were considered suitable for the study. The age range was 19–63 years, with a mean age of 33.3 years. The participants included 27 men and 74 women. There was a broad range of highest education level achieved (Grade 9 to Master's degree) and occupation (nurses, administrators, managers, cleaners, plumbers, waitresses and security personnel).

Table 1 depicts the distribution of the participant sample across the age groups, according to gender. As shown in Table 1, the participant sample included a broad range of age groups. The majority of the participants were women, as the sampling took place in a hospital, which is predominantly staffed by women (Pillay, 2009). The 30–39 year-old age group was the largest group.

**TABLE 1:** Distribution of participants by age and gender.

Gender	Age (years)						Grand total
	18–19	20–29	30–39	40–49	50–59	60–69	
Male	0	9	10	5	3	0	27
Female	1	17	20	20	12	4	74
Grand total	1	26	30	25	15	4	101

The participants’ first language use included Afrikaans (20%), Zulu (18%), Venda (16%), Sotho (11%), Pedi (10%), isiXhosa (3%), Ndebele (3%), Tsonga (3%), Tswana (2%), and Swati (1%) (10 of the 11 official South African languages, with the exception of English, which was not sampled) (Statistics South Africa, 2012). First-language speakers of isiXhosa were under-sampled because of the geographical location of the sampling. A broad range of educational levels was represented in the participant sample. Twenty-eight percent of the sample did not complete their secondary schooling; and 55% of the sample had no additional education other than high school.

In order to determine which of the wordlists (lists A, B or C) yielded the most favourable PTA/SRT correlation when testing a group of the South African ESL participants, the PTA/SRT correlation was determined as described below.

### Pure-tone average /speech recognition threshold correlation when using lists A, B and C

**FIGURE 1:**
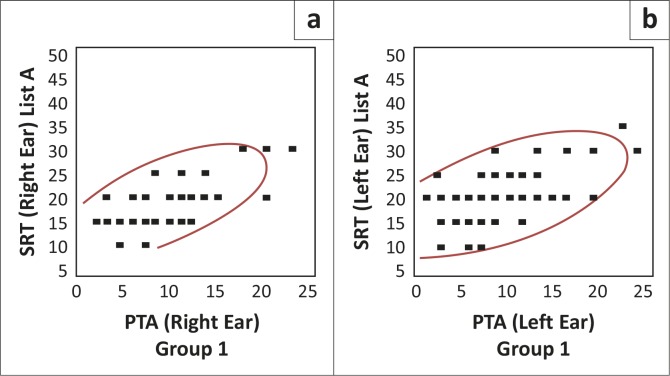
Pure-tone average/speech recognition threshold correlation for list A in group one (*N* = 54R; 51)

**FIGURE 2:**
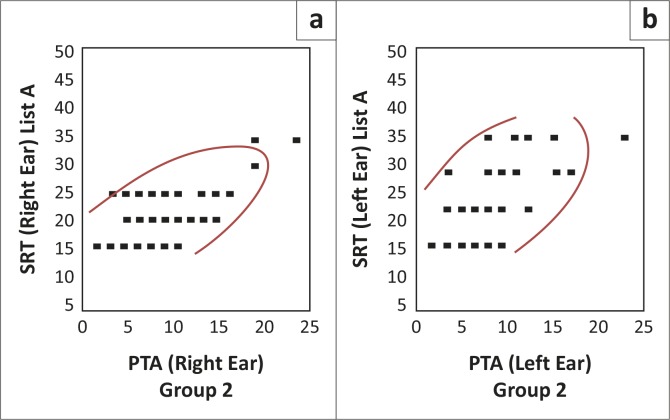
Pure-tone average/speech recognition threshold correlation for list A in group two (*N* = 46R; 46L)

**FIGURE 3:**
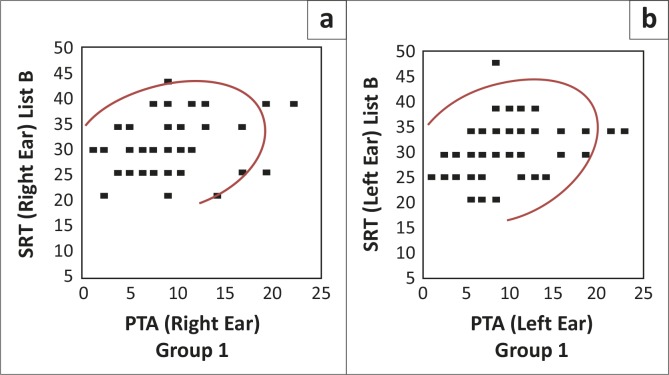
Pure-tone average/speech recognition threshold correlation for list B in group one (*N* = 54R; 51L).

**FIGURE 4:**
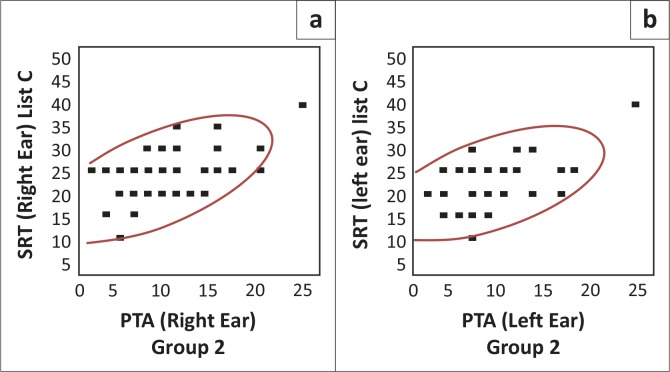
Pure-tone average/speech recognition threshold correlation for list C in group two (*N* = 46R; 46L)

A correlation matrix was calculated to measure the strength and direction of the relationship between two variables, namely PTA and SRT for lists A, B and C, which are depicted graphically in Figures 1–4 (H. Gerber pers. Comm., 08 July 2012 and 08 October 2012; Schiavetti & Metz, 2006). The scattergrams represent PTA for the right and left ears and SRT (lists A, B and C) for the right and left ears.

List A was also split for the two groups (groups one and two). The correlation strengths are described below. Figure 1 and Figure 2 are representations of the PTA/SRT correlations for groups one and two, respectively.

As may be seen in Figures 1–2, the scattergrams are densely clustered, with a similar pattern for groups one and two, for both the right and left ears (Schiavetti & Metz, 2006), which is suggestive of good inter-group reliability. The scattergram is clustered between 15 dB and 35 dB thresholds for SRT, with a corresponding range of 0 dB to 25 dB for PTA. A Pearson correlation analysis revealed a significant and positive, moderate-to-strong correlation relationship for list A. That is, as the variable of SRT tends to increase according to the increase of the variable of PTA, the relationship may be considered positive in all instances (Howell, 2009; Schiavetti & Metz, 2006).

Where ‘1’ denotes a perfect positive correlation and ‘0’ denotes no correlation (Howell, 2009), when compared to PTA for the right ear, list A (right ear) revealed a moderate-to-strong correlation of 0.63 (*N* = 54, *p* < 0.0001) for group one and 0.67 (*N* = 46, *p* < 0.0001) for group two. When compared to PTA for the left ear, list A (left ear) revealed a moderate-to-strong correlation of 0.58 (*N* = 51, *p* < 0.0001) for group one and 0.57 (*N* = 46, *p* < 0.0001) for group two. When the results for groups one and two were combined, list A (right ear) revealed a moderate-to-strong correlation of 0.65 (*N* = 100, *p* < 0.0001) (Schiavetti & Metz, 2006). When the results for groups one and two were combined, list A (left ear) revealed a moderate-to-strong correlation of 0.58 (*N* = 97, *p* < 0.0001) (Schiavetti & Metz, 2006). The relationship is considered moderate-to-strong (0.65 and 0.58 for the right and left ears, respectively) with the use of list A because of the high value of the correlation (Schiavetti & Metz, 2006).

For all conditions, the correlations between PTA and SRT were statistically significant for list A, at a 95% level of confidence, since the *p*-value is smaller than 0.05 (Schiavetti & Metz, 2006). In addition, there were no significant differences between groups one and two for list A, indicating a homogenous sample, no bias between groups and no advantage for either group, at a 95% level of confidence (Schiavetti & Metz, 2006).

The results indicate that the use of list A results in SRTs that have good correlation to PTA. This may be considered statistically significant. This result is as expected, as list A is considered to consist of the most familiar SAS words, according to Durrant (2006).

Figure 3 depicts a visual representation of the correlation strength of the PTA/SRT correlation when using list B.

As may be seen in Figure 3, the scattergrams are less densely clustered, with a similar pattern for the right and left ears (Schiavetti & Metz, 2006). The scattergram is clustered between 20 dB and 50 dB thresholds for SRT, with a corresponding range of 0 dB to 25 dB for PTA. This indicated much higher SRT responses when list B was used, despite the matched groups. A Pearson correlation analysis revealed a positive, moderate-to-weak correlation relationship for list B, for both the right (correlation of 0.30 [*N* = 54, *p* = 0.0266]) and left ears (correlation of 0.32 [*N* = 51, *p* = 0.0226]) (Howell, 2009; Schiavetti & Metz, 2006). This is indicative of a poor correlation with PTA when list B was used.

The correlations between PTA and SRT were statistically significant for list B, at a 95% level of confidence, since the *p*-value is smaller than 0.05 (Schiavetti & Metz, 2006). This result is as expected, because of the unfamiliar linguistic content and vocabulary of the words contained in list B. Many ESL South Africans have never been exposed to many of the words contained in list B and they are considered the most unfamiliar words, according to Durrant (2006).

Figure 4 depicts a visual representation of the correlation strength of the PTA/SRT correlation when using list C.

As may be seen in Figure 4 above, the scattergrams are densely clustered, with a similar pattern for the right and left ears (Schiavetti & Metz, 2006). The scattergram is clustered between 10 dB and 40 dB thresholds for SRT, with a corresponding range of 0 dB to 25 dB for PTA. A Pearson correlation analysis revealed a positive, moderate-to-strong correlation relationship for list C, for both the right (correlation of 0.63 [*N* = 46, *p* < 0.0001]) and left ears (correlation of 0.56 [*N* = 46, *p* < 0.0001]) (Howell, 2009; Schiavetti & Metz, 2006). The correlations between PTA and SRT were statistically significant for list C, at a 95% level of confidence, since the *p*-value is smaller than 0.05 (Schiavetti & Metz, 2006).The correlation value is high when list C was used. This result is as expected, as list C is considered to be the more familiar words of the original CID W-1 wordlist, according to Durrant (2006).

The results of the comparison of correlations obtained for lists A, B and C are presented in the next section.

### Pure-tone average/speech recognition threshold comparison of correlations for lists A, B and C

The values for the PTA/SRT correlations were tabulated for lists A, B and C for the whole group, as seen in Table 2.

**TABLE 2:** Summary of pure-tone average/speech recognition threshold correlation per list.

List administered	Right ear		Left ear	
	Correlation	Description	Correlation	Description
List A	0.65	Moderate-to-strong	0.58	Moderate-to-strong
List B	0.30	Moderate-to-weak	0.32	Moderate-to-weak
List C	0.63	Moderate-to-strong	0.56	Moderate-to-strong

As can be seen in Table 2, when determining the SRT correlation to the PTA, for both the right and left ears, the use of list A revealed a moderate-to-strong positive correlation, the use of list B revealed a moderate-to-weak positive correlation-, and the use of list C revealed a moderate-to-strong positive correlation (Schiavetti & Metz, 2006).

The correlation for each of the lists differs significantly from 0 at a 95% level of confidence since the *p*-value is smaller than 0.05 (Schiavetti & Metz, 2006). However, lists A and C revealed a higher absolute correlation than list B, indicating a stronger correlation for lists A and C.

## Discussion

The participants (*n* = 101) were considered a fair representation of the population of South Africa, with an age range of 19 to 63 years, both men and women, a broad range of education level and occupation and first language use. All of the participants were ESL speakers.

A large portion (42%) of the sample consisted of participants over the age of 40. Although all participants had hearing levels within normal limits (<26 dBHL), considerations in terms of age-related changes in auditory processing must be taken into account. Studies have shown an age-related difference in temporal processing skills (gap detection and interaural time differences) in older individuals (Pichora-Fuller & Souza, 2003). However, there is no evidence of a relationship between temporal resolution and performance in speech perception tasks. Although they are presented at low intensities, spondaic words are highly redundant and highly predictable, which excludes the factors of temporal cues on speech recognition tasks (Sreedhar et al., 2011). Therefore, the contribution of age may be considered negligible in terms of determining SRT.

A broad range of educational levels was represented in the participant sample. The participants are therefore representative of various groups of individuals, although this might have limitations as the generalisation may be compromised as a result of the relatively small sample size (Schiavetti & Metz, 2006). Because of exposure, language use and vocabulary, educational level has implications with regard to word familiarity (Song & Fox, 2008) and is thus a factor to be taken into account when considering the vocabulary content of unfamiliar wordlists. The sampled population may be considered to be a fair representation of the current educational levels in South Africa (Statistics South Africa, 2012).

PTA/SRT correlations were obtained for list A, B and C for the participants. PTA/SRT correlations of list A (0.65; 0.58), list B (0.30; 0.32) and list C (0.63; 0.56) were obtained for the right and left ears respectively. All the correlations for PTA/SRT were considered significant. The correlations for lists A and C were stronger than the correlation for list B, indicating a higher correlation for lists A and C. This is as expected, based on the familiar ratings of the wordlists (Durrant, 2006).

The PTA/SRT correlations for lists A and B differ significantly at a 99% level of confidence, yielding a stronger PTA/SRT correlation when list A was used. Lists A and C differ significantly at a 95% level of confidence, yielding a stronger PTA/SRT correlation when list A was used.

The correlation obtained for list A (0.65; 0.58) and list C (0.63; 0.56) is comparable to the correlation obtained by Khoza et al. (2008) when using the CID W-1 wordlist (0.61), a Tswana wordlist (0.62) and a digit wordlist (0.60), in their sample of university ESL students, as well as the correlation obtained by Ramkissoon et al. (2002) for the CID W-1 wordlist (0.63) for ESL speakers. However, the correlation obtained by Ramkissoon et al. (2002) for the digit wordlist was slightly stronger (0.71). This is evident of improved performance for digit testing, but only for the Ramkissoon *et al*. study (2002).

Interestingly, lists A and C yielded a correlation for the right ear which was stronger than the correlation for the left ear, despite the randomisation of test order. This may be related to right ear processing dominance for speech which may require further investigation in future studies (Bellis, 2003).

The use of list A yielded the highest PTA/SRT correlation. A statistically significant difference was found between the results obtained for PTA/SRT correlation with the use of the SAS wordlist and the CID W-1 wordlist, at a 0.05 level of statistical significance. Therefore, the use of the SAS wordlist yields a higher PTA/SRT correlation than the use of the CID W-1 wordlist, when performing SRT testing as part of the speech audiometry test battery on South African ESL speakers with normal hearing, or minimal hearing loss (<26 dBHL).

### Clinical implications

The use of the SAS wordlist may be employed tentatively when performing SRT testing as part of the speech audiometry test battery on a South African ESL speaker with normal hearing, or minimal hearing loss <26 dBHL, with the understanding that the wordlist has not been standardised at a national level.

Considering that the home language of the majority of audiology professionals in South Africa is either English or Afrikaans, whereas most South Africans speak an African language as their home language (Swanepoel, 2006), the use of an English wordlist which is familiar to the South African ESL population indicates a potential solution to the predicament faced when testing the SRT of ESL speakers, because of the implications of multilingualism on speech audiometry results (Von Hapsburg & Peña, 2002).

Although this is not an ideal solution to the predicament audiologists face when conducting speech audiometry in South Africa, in light of limited resources and the complex linguistic context of the country at present, this may be considered a reasonable solution which will yield more reliable results than using the lists which are currently available.

### Limitations

Although findings from the current study were significant, they should be interpreted with some caution because of the following limitations:

- The study may have been limited by the exclusive use of participants with normal hearing or minimal hearing loss <26 dBHL, as participants with different degrees of hearing loss were not included in the study.
- The participants were sampled from the province of Gauteng only and the results of the study may not be applicable to other provinces within South Africa, because of demographic differences in each province of South Africa (Alexander, 2000).
- The use of 5 dB increments was employed for the purpose of SRT determination. The use of 5 dB increments was preferred over the use of 1 dB or 2 dB increments, as this is applied more often in a clinical setting. The use of 1 to 2 dB increments may result in more accurate PTA/SRT correlations due to more specific SRT measurements.
- The quality of the compact disc recording was unfortunately substandard resulting in mean differences between PTA and SRT that are disproportionate for each of the wordlists, but the mean differences were equal for each list, which was accounted for in the statistical analysis. According to Di Berardino et al. (2010), the sensitivity of the VU meter should be adjusted according to the speech material levels prior to testing, in order to compensate for any differences. This was not identified prior to data collection.
- Lastly, the length of the SAS wordlist may be considered a limitation, because of the relative brevity of the SAS wordlist (18 words) in comparison to the recommended length of the CID W-1 (36 words) (Hirsh et al., 1952).

These limitations raise certain recommendations for future research.

### Recommendations

Firstly, future studies could include participants with hearing losses of various types, degrees and configurations. Secondly, participants from different regions of South Africa may be tested. Thirdly, future studies may use 1 dB or 2 dB steps for the purposes of determining SRT, as this allows for more precise information and correlation to be gleaned (Brandy, 2002). In addition, an improved recording quality would be strongly recommended and, should an improved recording quality be utilised, an equally proportioned improvement in PTA/SRT correlation would be expected for each of the wordlists, after which the sensitivity of the VU meter would be adjusted and specified accordingly (Di Berardino et al., 2010). Lastly, the evaluation of each of the spondaic words in the SAS wordlist should be conducted in terms of homogeneity of audibility, using a performance-intensity, or articulation gain curve, which gives information about the precision with which threshold can be obtained (Brandy, 2002). The SRT obtained using the SAS wordlist could also be compared with the SRT obtained when using a digit wordlist when testing ESL speakers in South Africa (Ramkissoon et al., 2002).

## Conclusion

The South African Spondaic wordlist contains the most familiar spondaic words to the South African population who use English as one of their multiple languages. The use of the SAS wordlist yields a statistically significantly stronger PTA/SRT correlation than the use of the CID W-1 when measuring SRT in the South African population with normal hearing or a minimal hearing loss < 26 dBHL, who use English as a second language.

## Appendix

**Table A1-1:** Available spondaic wordlists in terms of familiarity.

South African Spondaic (SAS) wordlist (Durrant, 2006)*	‘Less familiar’ CID W-1 wordlist (Durrant, 2006; Hirsh et al., 1952)	‘More familiar’ CID W-1 wordlist (Durrant, 2006; Hirsh et al., 1952)
LIST A	LIST B	LIST C
cellphone**	greyhound	schoolboy
bathroom	inkwell	sunset
sandwich	whitewash	grandson
building	mousetrap	toothbrush
township	duckpond	playground
dancing	sidewalk	cowboy
welcome	horseshoe	northwest
housewife	baseball	hotdog
lightning	stairway	mushroom
toothbrush	iceberg	hardware
basket	railroad	workshop
public	oatmeal	eardrum
workshop	drawbridge	headlight
suitcase	hothouse	birthday
birthday	daybreak	pancake
sunlight	airplane	armchair
homework	padlock	
sunshine	nutmeg	

*, Permission was expressly granted by the University of the Witwatersrand to use this wordlist for the current study.

**, ‘Cellphone’ is a South African English term for a mobile phone.
